# DAP12 Overexpression Induces Osteopenia and Impaired Early Hematopoiesis

**DOI:** 10.1371/journal.pone.0065297

**Published:** 2013-06-11

**Authors:** Geneviève Despars, Subramanya N. M. Pandruvada, Adrienne Anginot, Chantal Domenget, Pierre Jurdic, Marlène Mazzorana

**Affiliations:** Institut de Génomique Fonctionnelle de Lyon, Université de Lyon, Université Lyon 1, CNRS, Ecole Normale Supérieure de Lyon, Lyon, France; Georgia Regents University, United States of America

## Abstract

ITAM-bearing transmembrane signaling adaptors such as DAP12 and FcRγ are important players in bone homeostasis, but their precise role and functions are still unknown. It has been shown that osteoclast differentiation results from the integration of the RANK and of the DAP12 and FcRγ signaling pathways. DAP12-deficient mice suffer from a mild osteopetrosis and culture of their bone marrow cells in the presence of M-CSF and RANKL, fails to give rise to multinucleated osteoclasts. Here, we report that mice overexpressing human DAP12 have an osteopenic bone phenotype due to an increased number of osteoclasts on the surface of trabecular and cortical bone. This enhanced number of osteoclasts is associated with an increased number of proliferating myeloid progenitors in Tg-hDAP12 mice. It is concomitant with an arrest of B cell development at the Pre-Pro B/Pre B stage in the bone marrow of Tg-hDAP12 mice and important decrease of follicular and marginal B cells in the spleen of these animals. Our data show that the overexpression of DAP12 results in both increased osteoclastogenesis and impaired hematopoiesis underlining the relationship between bone homeostasis and hematopoiesis.

## Introduction

Bone remodelling, which is a constant ongoing process maintains bone integrity throughout life. During this process, bone resorption activity of osteoclasts and bone formation activity of osteoblasts achieve a dynamic balance [1] [2]. Under normal conditions, numerous local and systemic factors regulate bone remodelling such as increased or decreased mechanical loading, cytokines (Receptor Activator of NFκB ligand (RANKL), osteoprotegerin (OPG), Macrophage Colony Stimulating Factor (M-CSF), hormones (calcitonin, PTH, 1,25-dihydroxyvitamin D3, prostaglandin E2, leptin….), neuromediators (norepinephrine, dopamine, neurokinin B….) to which pro-inflammatory cytokines (IL-1β, IL-6, IL-11, IL-17, TNF-α), must be added under inflammatory conditions. Many bone diseases induce pathological imbalances between bone resorption and bone formation [3]. Osteoporosis, characterized by a decrease in bone mass and a degradation of bone microarchitecture, is mainly attributable to increased bone resorption activity of osteoclasts [4]. This is illustrated by different animal models such as the ovariectomized mouse model and, in humans, by the post-menopausal osteoporosis. On the other hand, osteopetrosis is characterized by an increased bone density and alterations of bone shape and structure. In murine models it relies either on the absence of osteoclast differentiation or on dysfunctional osteoclasts.

Osteoclasts are multinucleated hematopoietic cells resulting from the fusion of mononucleated precursors of the monocyte/macrophage lineage [5]. As such, they derive from early myeloid progenitors of the bone marrow (CMP; CFU-GM…) [6], [7] [8] and other late precursors such as monocytes, macrophages and dendritic cells [9] [10]. They display characteristic markers such as 5b isoform of the tartrate resistant acid phosphatase (TRAP5b), cathepsin K, calcitonin receptor, and integrin, but the ultimate signature of osteoclasts is their unique ability to degrade mineralized matrices [11]. Osteoclast differentiation requires the RANK/RANKL signaling pathway, as well as costimulatory pathways initiated by the transmembrane adaptor proteins DAP12 and FcRγ which contain an immunoreceptor tyrosine-based activation motif (ITAM) in their intracytoplasmic domain [12]. RANK and ITAM signals merge to cooperatively stimulate activation of NFATc1, the master transcription factor in osteoclastogenesis [13]. Depending on cell type, DAP12 is associated with multiple cell surface activating receptors in hematopoietic cells. For example, in osteoclasts, it is associated with TREM-2 and SIRPβ1 [12]. Besides osteoclasts, DAP12 and FcRγ are predominantly expressed in cells of the myeloid lineage (DC, monocytes, macrophages, microglial cells) in which they participate in multiple biological functions [14]. DAP12 is weakly expressed at the surface of the cells of the adaptative immunity such as B and T lymphocytes [15] and is preferentially expressed by these cells with an activated phenotype, often related to inflammatory conditions [16].

In humans, mutations of genes coding for DAP12 or its associated receptor TREM-2 lead to the PLOSL disease (polycystic lipomembranous osteodysplasia with sclerosing leucoencephalopathy), also known as Nasu-Hakola disease. Patients suffer from spontaneous fractures and presenile dementia resulting in premature death [17], [18]. Studies on mice, either deficient in DAP12 (DAP12^−/−^ mice) or bearing a mutated form of the adaptor that prevents signaling (KΔ75 mice), have shown that, depending on the genetic background of the animals and/or culture conditions, osteoclast precursors are either unable to differentiate *in vitro* into multinucleated osteoclasts in the presence of M-CSF and RANKL or form only few small multinucleated cells [19], [20], [21], [22]. Surprisingly, in DAP12^−/−^ mice osteoclasts were present although non resorbing and the animals present only a mild osteopetrosis. This discrepancy between *in vitro* and *in vivo* observations has been explained by a study of the double mutant DAP12^−/−^ FcRγ^−/−^ mice and the *in vitro* coculture of osteoclasts and osteoblasts. These results suggest that FcRγ is able to compensate for the absence of DAP12. It has been recently shown that osteoblasts, by synthesizing collagen, provide an essential signal through FcRγ and its co-receptor OSCAR (osteoclast-associated receptor in the absence of DAP12 *in vivo* [23]. Furthermore, our previous work suggests that lymphocytes can also partially compensate for the lack of DAP12 *in vitro* and *in vivo* [24]. Finally, DAP12 exerts a protective effect on ovariectomy-induced bone loss in the trabecular long bones which is not observed in the vertebrae and the cortical bone [25]. This leads to the idea that the effect of ITAM adaptor signaling, although critical for bone remodeling, depends on the bone microenvironment and pathological conditions. In order to understand better the role of DAP12 in bone, we analysed the bone phenotype of the DAP12 overexpressing mice [26]. Here we show that DAP12 overexpression induces osteopenia related to an increased number of osteoclasts. Furthermore, we confirm disturbances of hematopoiesis in these animals, that we suspect to be related to disturbances of the bone microenvironment (niches).

## Materials and Methods

### Animals

Transgenic mice expressing the human DAP12 cDNA (Tg-hDAP12) under the control of the Major Histocompatibility Class 1 promoter with 11 or 30 copies of the transgene have been previously described [26]. The transgenic animals used in this work were Tg-hDAP12-11 (with eleven integrated transgene copies). They were a generous gift of E. Tomasello and E. Vivier (CIML, Marseille, France). Twelve backcrosses with C57BL6 mice were done. Mice were housed under aseptic conditions with 12-hours light cycle and given tap water and food *ad libitum* (Plateau de Biologie Expérimentale de la Souris, Ecole Normale Supérieure de Lyon, Lyon, France). Genotyping was also done at the Plateau de Biologie Expérimentale de la Souris (Ecole Normale Supérieure de Lyon).

### Ethics Statement

All steps were performed to limit suffering in all experiments involving our study animals. The study was approved by the Ethical Committee of the Ecole Normale Supérieure de Lyon.

### Flow Cytometry Analyses

Flow cytometry analysis was performed on spleen and bone marrow cells of 10-week-old female mice (four WT animals and four Tg-hDAP12). Bone marrows were flushed and spleens were dissociated in FACS buffer (PBS, 1% serum (Biowest), 2 mM EDTA), then filtered through a 100 µm cell strainer (Falcon). Red cells were lysed by a ten-minute incubation at room temperature in 0.15 M ammonium chloride with 0.017 M potassium carbonate (ACK buffer). Single cell suspensions of spleen or BM cells were stained using standard protocols for flow cytometry. The following conjugated antibodies were used: PE-Cy5-conjugated anti CD19 (clone 1D3), eFluor 450-conjugated anti CD45R/B220 (clone RA3-6B2), A647-conjugated anti IgD (clone 11–26), PE-Cy5.5-conjugated anti IgM (clone II/41), APC-Alexa750-conjugated anti CD11b (clone M1/70), PE-conjugated anti Ly6G (clone RB6-8C5) were from eBioscience; PE-Cy7-conjugated anti CD25 (clone PC61) and FITC-conjugated anti Ly6C (clone AL-21) were from BD Bioscience. FACS analysis was performed on a LSRII cytometer (BD Biosciences). Data were analyzed with FlowJo or DiVa softwares (BD Biosciences) and represented, when required, with the logical display 20.

### Bone Analysis Using High-Resolution Microtomography

Tibias dissected from 1-, 3- and 6-month old mice were fixed in 4% (w/v) paraformaldehyde in PBS, ethanol-dehydrated and scanned with a high-resolution micro-Computed Tomography (µCT) (Viva CT40; Scanco Medical, Bassersdorf, Switzerland), while maintained in ethanol during image acquisition. Data were acquired at a 55 KeV energy, with a 145 mA current for a 10 µm cubic resolution. Three-dimensional reconstructions were generated with the following parameters: Sigma, 1.2; Support, 2; Threshold, 160 (spongiosa) or 280 (cortex) for the samples. Cortical thickness and tissue mineral density were calculated by integrating the values for each transverse section of a set of 100 chosen in the midshaft area. Tissue mineral density was derived from the linear attenuation coefficient of thresholded bone after precalibration of the apparatus for the acquisition voltage chosen. The bone volume fraction of trabecular metaphysis (VOX BV/TV) was measured on a set of 80 sections under the growth plate, within the secondary spongiosa. Trabecular thickness (Tb.Th), trabecular number (Tb.N), trabecular separation (Tb.Sp), and structure model index (SMI) were calculated without assuming a constant model, as previously described [27]. SMI estimates the plate-rod characteristics of a structure; its value is 0 for an ideal plate, and 3 for an ideal rod, with intermediate values reflecting the volume ratio between rods and plates. The parameters of porosity, cortical thickness (Ct.Th.), cross-sectional or total area (Cr.Sc.Ar.), marrow area (Ma.Ar.) and bone area (Ct.Ar.) were calculated using a dedicated software (Laboratoire du Tissu Osseux et Contraintes Mécaniques, Université Jean Moulin, St-Etienne, France).

### Histomorphometry

Calcified posterior limbs of 3-month-old female mice were embedded in methylmethacrylate as previously described [28]. Seven-µm thick methylmethacrylate sections were stained for TRAP positive osteoclasts. Bone cellular and macroscopic measurements were done with a DMLB microscope (Leica, Nanterre, France) connected to a 3CCD colour video DXC-390 camera (Sony, France) and using the OsteoMeasure Analysis System (Osteometrics, Decatur, USA). For bone formation rate measurement mice were injected twice with calcein (Sigma Aldrich, Lyon, France) at a four-day interval prior to sacrifice.

### DPD/Creatinine Assays

Evening urine samples were collected from 3-month-old female mice and 6-month-old male mice and kept frozen until quantification. Deoxypyridinoline (DPD) and creatinine were quantified by ELISA using the MetraDPD Enzyme ImmunoAssay kit and the MetraCreatinine Assay kit (Quidel Corporation, San Diego, CA, USA).

### RANKL and OPG Immunoassays

400 µl of blood were colleted by retro-orbital punction in five 3-month-old WT and Tg-hDAP12 female mice. Blood samples were allowed to coagulate two hours at room temperature. They were then centrifugated 20 min., 2000 g at room temperature. The supernatants (sera) were frozen at –80°C until they were used for the ELISA assays. Amounts of OPG and RANKL present in the sera were measured using Quantikine® ELISA Mouse OPG/TNFRSF11B Immunoassay and Quantikine® ELISA Mouse TRANCE/RANKL/NFSF11 Immunoassay (R&D Systems Europe, Lille, France), following manufacturer's instructions. The optical density (OD) of the samples in duplicate was determined at 450 nm using a microplate reader with wavelength correction set at 540 nm and used to calculate the amounts of RANKL and OPG in the sera in reference to standard curves.

### Generation of Osteoclasts from Spleen and Bone Marrow Cultures

Spleens and posterior limbs were collected from mice of stated ages. Cell suspensions were prepared by crushing the spleen over nylon mesh (BD Falcon, Le-Pont-de-Claix, France) or flushing bone marrow cells using complete medium (α-MEM) (Invitrogen, Cergy Pontoise, France) supplemented with 10% fetal bovine serum (Biowest, Nuaillé, France), 100 U/ml penicilline, 100 µg/ml streptomycine and 2 mM L-glutamine (Invitrogen)). Mononuclear cells isolated using Lymphocyte Separation Medium (EuroBio, Courtaboeuf, France) were seeded in complete α-MEM medium supplemented with 25 ng/ml mouse Receptor-Activator of NF-κB ligand (RANKL; R&D Systems, Lille, France) and recombinant mouse macrophage-colony stimulating factor at 25 ng/ml (M-CSF; R&D Systems, Lille, France). Bone marrow or spleen mononuclear cells from the same WT or Tg-hDAP12 animal, were seeded at 12.5 x 10^3^ or at 78×10^3^ cells/well respectively, in triplicate in 96-well plates, with a change of medium every second day. For the kinetic studies of spleen cell-derived osteoclastogenesis, cells were seeded in triplicate at 78×10^3^/well of 96-well plate. They were fixed every two days and stained for tartrate resistant acid phosphatase (TRAP) activity using the leukocyte acid phosphatase kit from Sigma Aldrich, following the manufacturer’s instructions. Pictures were taken using an Axiovert 25 (Zeiss, Le Pecq, France) connected to a Leica DC 180 colour camera (Leica, Nanterre, France). All TRAP-positive cells with ≥3 nuclei present in each well were counted at each time point, using an IMT2 inverted microscope (Olympus, Rungis, France) or a Nikon Eclipse TL100 (Nikon France S.A., Champigny sur Marne, France).

For the study concerning the response of bone marrow and spleen cells to RANKL, splenic cells of three 3-month-old WT females or three 3-month-old Tg-hDAP12 female mice were used. Splenic and bone marrow cells were plated in triplicate in wells of 96-well plates in the α-MEM medium described previously and containing either 20, 30, 40, 50 or 100 ng/ml of RANKL (bone marrow cells) or 20, 30, 50 or 100 ng/ml of RANKL (spleen cells). Splenic WT cells were plated at 3×10^5^ cells/well whereas splenic Tg-hDAP12 cells were plated at 10^5^ cells/well. Bone marrow WT and Tg-hDAP12 cells were plated at 0.15×10^5^ cells/well. Spleen cells were fixed at day 6 and their bone marrow counterparts were fixed at day 4. They were then stained for TRAP activity. Osteoclasts with nuclei ≥3 were counted.

### CFU-GM and CFU-M Assays

#### CFU-GM assays

Spleens from 3-month-old WT and Tg-hDAP12 female mice were dissociated in previously described complete α-MEM culture medium. As for flow cytometry studies, red cells were lysed in ACK buffer. To determine CFU-GM, triplicate cultures were done by seeding 2×10^5^ spleen cells in 1 ml of methycellulose in wells of 24-well plate (Methocult 3534, Stem Cell Technologies, Vancouver, Canada) containing SCF, IL-3 and IL-6 according to manufacturer's instructions. WT and Tg-hDAP12 colonies were enumerated using an inverted microscope after seven days of incubation at 37°C, picked and diluted in culture medium. Part of the cells were submitted to centrifugation using Shandon Cytospin 4 (Thermo Electron Corporation). Spots of cells were then fixed in methanol and stained with Wright-Giemsa. The rest of the cells was submitted to flow cytometry. We used the following conjugated antibodies: PE-conjugated anti CD11b (clone M1/70), Alexa Fluor488-conjugated CD45R/B220 (clone RA3–6B2), PE-conjugated anti Gr-1(Ly6-G (GR-1) and Ly6-C (clone RB6–8C5) from BD-Pharmingen.

#### CFU-M assays

Spleens and bone marrows from 3-month-old WT and Tg-hDAP12 female mice were prepared as described above. Bone marrow cells were seeded at 0.2×10^4^, 1× 10^4^ and 2×10^4^ cells/plate ∅ 35 mm in 2 ml of methylcellulose (Methocult without cytokines, Stem Cell Technologies, Vancouver, Canada) supplemented with 30 ng/ml M-CSF. Concomitantly, spleen cells were seeded at 0.2×10^6^, 0.6×10^6^ and 1× 10^6^ cells/plate ∅ 35 mm in 2 ml of the medium previously described. Colonies were numerated 8 days after seeding in methylcellulose.

### Proliferation of Spleen Osteoclast Precursors *in Vitro*

Mononucleated spleen cells of 3-month-old female mice were isolated using Lymphocyte Separation as mentioned before. They were seeded in complete α-MEM medium supplemented with 25 ng/ml mouse recombinant RANKL and 25 ng/ml of recombinant mouse M-CSF as previously described (3 x 10^5^ cells/well in triplicate in 96-well plate) with a change of medium every second day. Quantification of the proliferation of cultured spleen osteoclast precursors was based on the measurement of BrdU incorporation during DNA synthesis in proliferating cells. For this purpose a colorimetric immunoassay was used to detect the incorporated BrdU (Cell proliferation ELISA BrdU (colorimetric)), Roche applied science) in accordance with manufacturer's instructions. Briefly, at each time point of the study, cells were incubated three hours with BrdU at 37°C. After removal of BrdU, cells were fixed and denatured 30 min. at room temperature, treated with peroxydase-conjugated anti- BrdU during 90 min at room temperature. Finally, after incubation with peroxydase substrate, the reaction product was quantified by measuring the absorbance at 370 nm with reference wavelength at 492 nm using a multiwell ELISA reader. Three 3-month-old wild type and Tg-hDAP12 female mice were used for the study.

### Generation of Osteoblasts from Bone Marrow Culture and Bone Nodule Quantification

Bone marrow was obtained from 3-month-old young adult wild type and Tg-hDAP12 female mice. Femurs and tibias were excised aseptically, cleaned of soft-tissue. Both epiphyses were cut off and the marrow cavities were flushed using 5 ml of culture medium expelled from a 21G needle. Freshly isolated whole bone marrows were plated at a density of 10^7^ cells in 100 cm^2^ dish and cultured for 3 days undisturbed. Thereafter complete medium was renewed every 2 days with MEM containing nucleotides, 10% FBS, antibiotics, ß sodium-glycerophosphate (10 mM) and ascorbic acid (50 µg/ml) for 7 days. Cells were trypsinized and plated at a density of 10^6^ cells in 100 cm^2^ dish and cultured in the above medium for 14 days. For quantification of bone formation, wells were fixed and stained for alkaline phosphatase activity (Kit Sigma ALP #086) and for mineralization using von Kossa. Plates were air-dried and mineralized colonies (brown-black coloured colonies) were counted using an ocular grid.

### Co-cultures of Bone Marrow-derived Osteoblasts and Spleen Cells

Freshly isolated bone marrow cells from 3-month-old WT or Tg-hDAP12 female mice were plated, as described above, in MEM containing nucleotides, 10% FBS, antibiotics and ascorbic acid (50 µg/ml) for 7 days. Cells were then trypsinized and plated at a density of 35×10^3^ cells/well of 6-well plates. The day after, cells, isolated as previously mentioned from the spleen of 3-month-old WT or Tg-hDAP12 female mice, were added to the bone marrow-derived osteoblasts at a density of 12×10^5^ cells/well. Culture medium was changed to support osteoclast differentiation: MEM without nucleotides, 10% FBS, antibiotics and ascorbic acid (50 µg/ml) supplemented with 1α,25-dihydroxyvitamin D3 at 10^−8^ M. For each of the four conditions, four wells of co-culture have been prepared and three other wells were maintained without addition of spleen cells. After six days of culture, co-cultured cells were fixed, stained for TRAP activity and the total number of generated osteoclasts was counted in each of the four wells. The cells in the three control wells, without spleen cells, were fixed and stained for alcaline phosphatase using a Leukocyte Alcaline Phosphatase kit (Sigma Aldrich, Saint-Quentin Fallavier, France).

### Reverse Transcription PCR

Total RNA was extracted with TRIzol^®^ (Invitrogen, France) reagent from mouse bone marrow cultures at different times of culture: day 3, day 7 and day 14. Samples of total RNA (1 µg) were reverse-transcribed using iScript cDNA synthesis kit (Bio-Rad). Osteoblast associated markers were amplified by PCR using primers specific for mouse alkaline phosphatase (ALP) and osteocalcin (OCN). The PCR reaction mixture contained cDNA (2 µl), 1 µl dNTP mix (10 mM), 10 µl of GoTaq Flexi buffer, 25 mM MgCl2 solution, 25 pmol primers and 5 U of Taq polymerase (Promega). PCR was done for 24 cycles (94°C for 1 min, 55°C for 1 min, 72°C for 1 min, and with a final elongation step of 7 min at 72°C) for L32 (400 bp) and 25 cycles for OCN (276 bp), and ALP (405 bp). The expression of human DAP12 in osteoblasts was measured using the same RT-PCR conditions. The PCR products were electrophoresed using 1.2% agarose gels. Images were photographed and band intensities were measured using ImageJ software. Gene expression was normalized to the corresponding L32 (ribosomal protein 32) house keeping gene values.

Primers were: ALP upstream: 5′-CCC GAATCC TTA AGGGCC AG-3′; ALP downstream: 5′-TATGCGATGTCCTTGCAGC-3′; OCN upstream: (5′-TGA CAA AGC CTT CAT GTC CA-3′; OCN downstream: (5′-GAG AGG ACA GGG AGG ATC AA-3′; L32 upstream: 5′-CAT GGC TGC CCT CCG GCC TC-3′; L32 downstream: 5′-CAT TCT CTT CGC TGC GTA GCC-3′; hDAP12: upstream: 5′-ATGGGGGGACTTGAACCCTGC-3′; hDAP12 downstream: 5′-GTATCATGTTGCTGACTGTCA-3′; mHPRT upstream: 5′- GCTGGTGAAAAGGAC CTC-3′; mHPRT downstream: 5′-CACAGGACTAGAACACCTCGT-3′.

### Statistics

Results are expressed as mean ± standard error (SE) or absolute error (AE). Depending on the sample number (*n*), statistical test were done using either the Student’s t test with bilateral distribution and equal variance (results were considered significant for *p*<0.05 and labeled with *), or the Mann-Whitney rank sum test (results were considered statistically highly significant when *p*<0,001 (***); significant when *p*<0,01 (**); marginally significant when *p*<0,05.

## Results

### Early Onset of Osteopenia at Three Months in Tg-hDAP12 Female Mice

**Figure 1:**
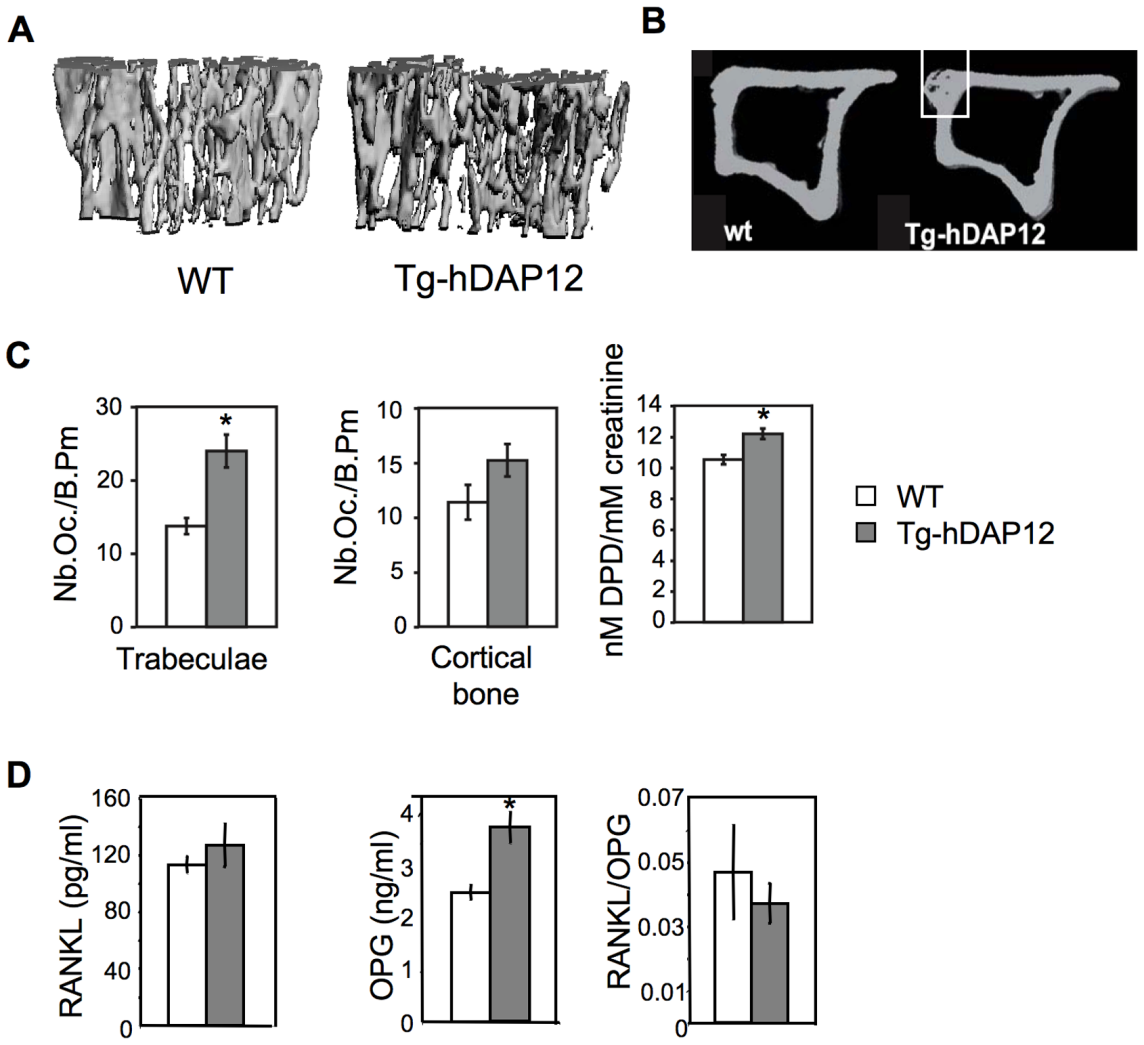
Bone phenotype of 3-month-old Tg-hDAP12 female mice. (A and B) 3D images of trabecular and cortical bone of proximal tibias. In B the white insert shows the increased porosity of the cortical bone of Tg-hDAP12 mice (groups of n = 8). (C) Left and middle panel show the number of osteoclasts per bone perimeter (Nb.Oc./B.Pm.) in the trabeculae and the cortical bone (n = 6). Results are means ± SE. *: p<0.05. Right panel shows the ratio of urinary DPD (nM) versus creatinine (mM). Groups of n = 8, results are means ± SE. *: p<0.05. (D) Quantification of RANKL and OPG in the sera of WT and Tg-hDAP12 female mice aged 3 months using mouse RANKL and OPG immunoassays (left and middle panels respectively). Ratio of RANKL/OPG (right panel). Five sera of each category (WT and Tg-hDAP12) were collected for the study. Results are expressed as means ± SE.

We examined the bone phenotype of Tg-hDAP12 female and male mice *in vivo*. Two age groups were used during the course of mouse development: 1-month-old pre-pubescent mice and sexually mature 3-month-old mice. Tibias from 1-month-old female and male mice were analysed by µCT. Similar values between control and transgenic animals were obtained for the dynamic trabecular parameters of BV/TV, connectivity, number of trabeculae, trabecular separation and thickness between WT and Tg-hDAP12 female mice (Table 1). The situation was quite different in the tibias of the 3-month-old female mice investigated using µCT (Table 2). Quantification of bone parameters revealed an obvious osteopenia which was evidenced by decreased bone volume for proximal tibias (-28.5%) and distal femurs (-35.2%, data not shown). Decrease in tibia trabecular connectivity (-49.3%) resulted from decreased trabecular number (Tb.N.), increased trabecular separation (Tb.Sp.) and trabecular thinning (Tb.Th.). Similar results were obtained from femurs (data not shown). 3D images of proximal tibias of 3-month-old female illustrated low bone mass, with thinning of trabeculae and decreased trabecular connectivity (Fig. 1A). Thinning of tibial cortices (Ct.Th.) as well as increase of porosity were also evident in Tg-hDAP12 mice (Fig. 1, B). Bone loss was associated with an increase in the number of osteoclasts per trabecular bone perimeter as well as in the number of osteoclasts on the endosteal surface of cortical bone for Tg-hDAP12 compared to WT mice (Fig. 1, C, left and middle diagrams), indicating that bone was degraded on both trabecular and endosteal surfaces. The increased number of osteoclasts resulted in an increased urinary excretion of desoxypyridinoline (DPD), a degradation product of type I collagen used to measure osteoclast function *in vivo*. A significant increase of 16% in DPD/creatinine ratio was observed for Tg-hDAP12 female mice compared to WT mice (Fig. 1, C, right diagram). The level of RANKL was not statistically different in the sera of both WT and transgenic animals (Fig. 1, D, left panel) indicating that the increased number of osteoclasts present in the bone of Tg-hDAP12 mice was not related to an increased level of RANKL. In contrast and surprisingly, in the context of osteopenia, the level of OPG was increased (1.5-fold) in the sera of Tg-hDAP12 mice (Fig. 1, D, middle panel). This led to a decreased RANKL/OPG ratio in Tg-hDAP12 mice (Fig. 1, D, right panel). Since bone resorption and formation are coupled, we therefore quantified bone formation rates using calcein standard double-labeling protocol. No differences in bone formation rate were observed between WT (263.57±36.62 µm^3^/µm^2^/year) and Tg-hDAP12 (239.92±36.99 µm^3^/µm^2^/year) 3-month-old female mice. These results indicated that the observed osteopenia in females at 3 months was related to increased bone degradation and not to a decreased bone formation.

**Table 1:** Trabecular bone parameters for tibias from 1-month-old mice.

	Female		Male	
Parameters	WT	Tg-hDAP12	WT	Tg-hDAP12
% BV/TV¶	5.6±0.2	5.8±1.1	4.6±0.3	4.2±0.5
Conn.D.(1/mm^3^)	26.3±3.9	36.2±15.2	21.9±2.5	18.9±3.7
Tb.N.(1/mm)	4.0±0.1	4.2±0.4	3.5±0.3	3.4±0.3
Tb.Th. (µm)	34.8±0.6	33.9±1.4	34.1±0.3	33.9±0.5
Tb.Sp. (µm)	248.4±7.9	252.9±22.0	296.6±21.9	306.4±22.1

¶ Abbreviations used: BV/TV: bone volume over total volume; Conn.D.: connectivity; Tb.N.: number of trabeculae; Tb.Th.: trabecular thickness; Tb.Sp.: trabecualr spacing. For each group n = 6–8.

**Table 2:** Trabecular and cortical parameters for tibias from 3-month-old mice.

	Female			Male	
Parameters	WT	Tg-hDAP12	% change	WT	Tg-hDAP12
% BV/TV¶	14.85±0.91	10.61±0.74^†^	–28.5	22.33±1.40	23.41±0.05
Conn.D.(1/mm^3^)	92.41±9.70	46.81±4.46^†^	–49.3	135.83±12.40	127.76±9.15
Tb.N.(1/mm)	4.50±0.13	3.84±0.13^†^	–14.6	5.58±0.17	5.34±0.13
Tb.Th. (µm)	52.41±0.89	49.63±1.97	–5.3	57.76±1.98	60.89±1.18
Tb.Sp. (µm)	216.29±6.73	260.15±10.98^†^	+20.3	164.95±5.42	174.16±4.94
Ct.Th. (µm)	168.38±3.66^†^	144.75±8.29^†^	–14	168.10±4.55	156.40±2.40
Porosity %	4.24±0.55	6.19±0.88		2.68±0.14	2.64±0.09

¶ Abbreviations used: BV/TV: bone volume over total volume; Conn.D.: connectivity; Tb.N.: number of trabeculae; Tb.Th.: trabecular thickness; Tb.Sp.: trabecualr spacing; Ct.Th.: cortical thickness. ^†^indicates significantly different results, (p<0.05 versus corresponding parameters in WT females). Results expressed as means ± standard error, for each group n = 6–8. Increases or decreases in female trabecular and cortical parameters are indicated as "% change".

Males of the same age did not present osteopenia. Similar values for trabecular bone parameters were obtained for the tibias of WT and Tg-hDAP12 male mice in sharp contrast with the osteopenia observed in the tibias of 3-month-old Tg-hDAP12 female mice (Table 2).

### Late Onset of Osteopenia at Six Months in Tg-hDAP12 Male Mice

In the absence of bone phenotype in 3-month-old transgenic males, we tested if the onset of osteopenia was delayed in males and we observed their bone phenotype at 6 months (Table 3). Trabecular bone from the tibias and femurs (data not shown) declined by 15.2 and 21% respectively, in Tg-hDAP12 male mice and connectivity was 23.4% less than in the WT animals. The decrease in tibial BV/TV was characterized by a decrease in the number of trabeculae and an increase in trabecular separation, while trabecular thickness remained unaffected. Similar results were obtained for the femurs (data not shown). There was also a decrease in cortical thickness (-9.2%) for tibias of 6-month-old Tg-hDAP12 male mice compared to WT. DPD measurements in the urine of 6-month-old male mice revealed a net increase in urinary DPD/creatinine ratio in Tg-hDAP12 compared to WT male mice at 6 months (+28%). In summary, DAP12 overexpression resulted in an age-and sex-dependent bone phenotype: a rapid onset of osteopenia in post-puberty female mice, whereas osteopenia appeared only in older male animals.

**Table 3:** Trabecular and cortical bone parameters for tibias from 6-month-old male mice.

Parameters	WT	Tg-hDAP12	% change
% BV/TV¶	27.45±1.14	23.27±1.33^†^	–15.2
Conn.D.(1/mm^3^)	127.6±6.16	97.77±3.71^†^	–23.4
Tb.N.(1/mm)	5.38±0.13	5.04±0.07^†^	–6.3
Tb.Th. (µm)	63.40±1.37	62.77±1.90	–1
Tb.Sp. (µm)	168.87±5.19	182.57±2.97^†^	+8.1
Ct.Th. (µm)	169±5.69	153.40±0.04^†^	–9.2
Porosity %	3.19±0.34	2.99±0.16	

¶ Abbreviations used: BV/TV: bone volume over total volume; Conn.D.: connectivity; Tb.N.: number of trabeculae; Tb.Th.: trabecular thickness; Tb.Sp.: trabecualr spacing; Ct.Th.: cortical thickness. ^†^indicates significantly different results, (p<0.05 versus corresponding parameters in WT females). Results expressed as means ± standard error, for each group n = 10. Increases or decreases in female trabecular and cortical parameters are indicated as "% change".

To further confirm these *in vivo* results we undertook an *in vitro* analysis of both osteoblastogenesis and osteoclastogenesis in female mice.

### Overexpression of DAP12 has no Effect on Differentiation and Bone Formation Activities of Bone Marrow Osteoblasts *In* Vitro

**Figure 2:**
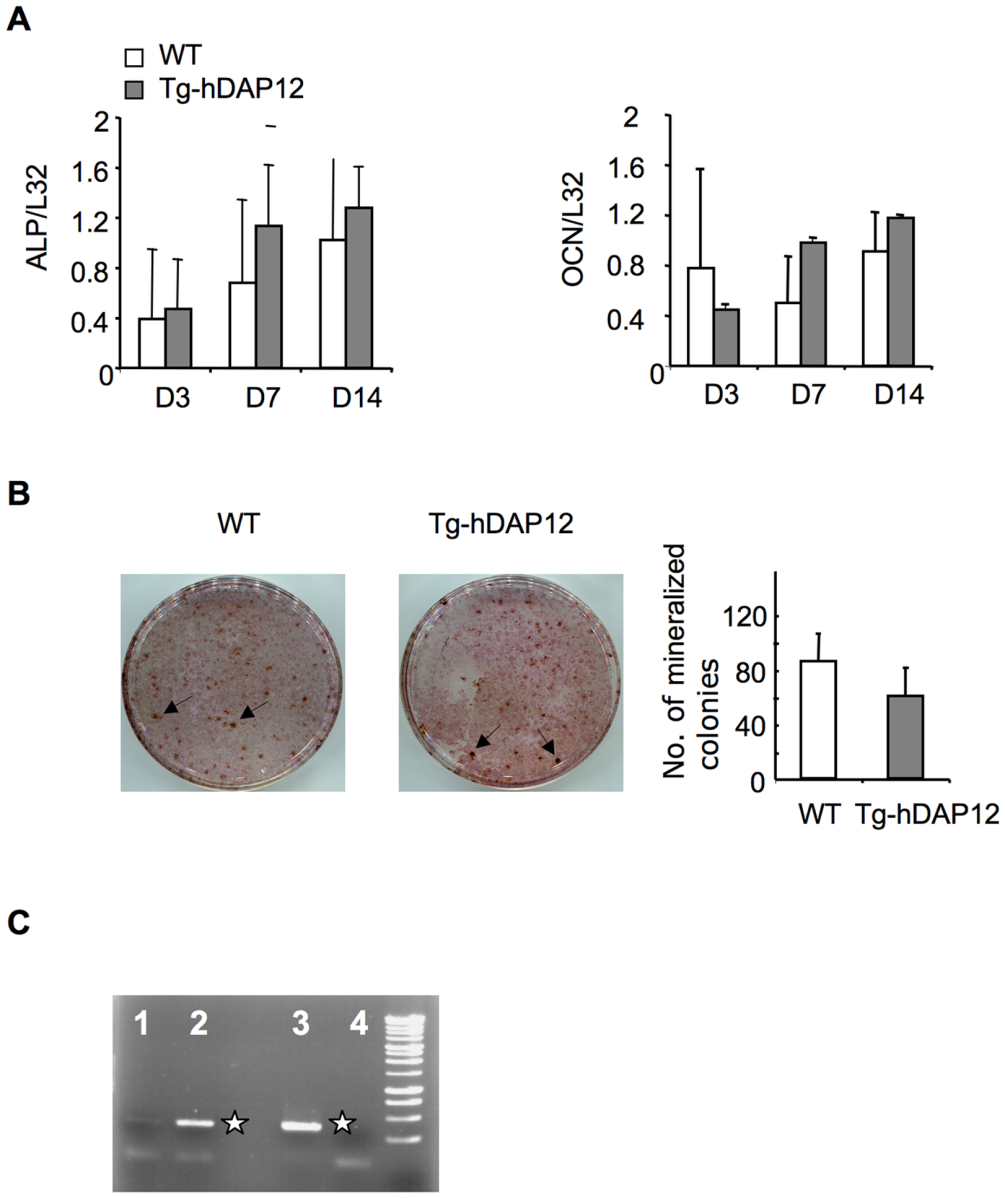
Osteoblastogenesis and osteoclastogenic activities of osteoblasts in bone marrow cell cultures from 3-month-old Tg-hDAP12 female mice. Osteoblasts were classically obtained after treatment of bone marrow cells with ß sodium-glycerophosphate (10 mM) and ascorbic acid (50 µg/ml) for 7 days. Total RNA was extracted at different times of culture: day 3, day 7 and day 14. (A) Gene expression of osteoblast-associated markers: alkaline phosphatase (ALP), osteocalcin (OCN), measured by RT-PCR at the indicated times of culture. Gene expression was normalized to the L32, house-keeping gene values. Results are means of two independent experiments ± SE. (B) Mineralized colonies obtained from osteoblasts generated from bone marrow. Colonies doubly-stained with alkaline phosphatase and von Kossa appear as black dots. Results are plotted as the mean number of nodules ±SE of three wells for 3-month-old WT and Tg-hDAP12 female mice and were representative of three independent experiments. (C) Expression of human DAP12 transgene in 7-day-old bone marrow-derived osteoblasts using RT-PCR. 1: WT osteoblasts; 2: Tg-hDAP12 osteoblasts. 3: hDAP12 expression in human monocyte-derived osteoclasts used as control; 4: RT-PCR without template. White stars indicate the 373 bp PCR fragment corresponding to hDAP12. One experiment is shown, representative of three independent experiments. Grey bars: Tg-hDAP12 mice; white bars: WT mice.

When osteoblasts were cultured from bone marrow cells of 3-month-old WT or Tg-hDAP12 female mice in the presence of glycerophosphate and ascorbic acid up to 21 days, a normal osteogenic development occurred in both WT and Tg-hDAP12 cultured bone marrow cells. This is shown by an increased expression of alkaline phosphatase and osteocalcin from day 3, when osteoblastic precursors were proliferating, to day 7, during the phase of matrix maturation and day 14, at the beginning of the mineralization phase [29]. At each time point (D3, D7, D14) there was no statistically-significant difference (*p values* >0.05) in the expression of the two genes during osteoblastogenesis in Tg-hDAP12 and WT mice (Fig. 2, A). Confirming RT-PCR data, alkaline phosphatase and von Kossa double staining revealed similar numbers of mineralized colonies (Fig. 2, B) indicating that DAP12 overexpression did not modify osteoblast differentiation and osteoblast-mediated bone formation activities. It has to be noticed that the hDAP12 transgene is clearly overexpressed in bone marrow osteoblasts of Tg-hDAP12 mice (Fig. 2, C).

### Accelerated Osteoclastogenesis in Tg-hDAP12 Spleen Primary Cultures

**Figure 3:**
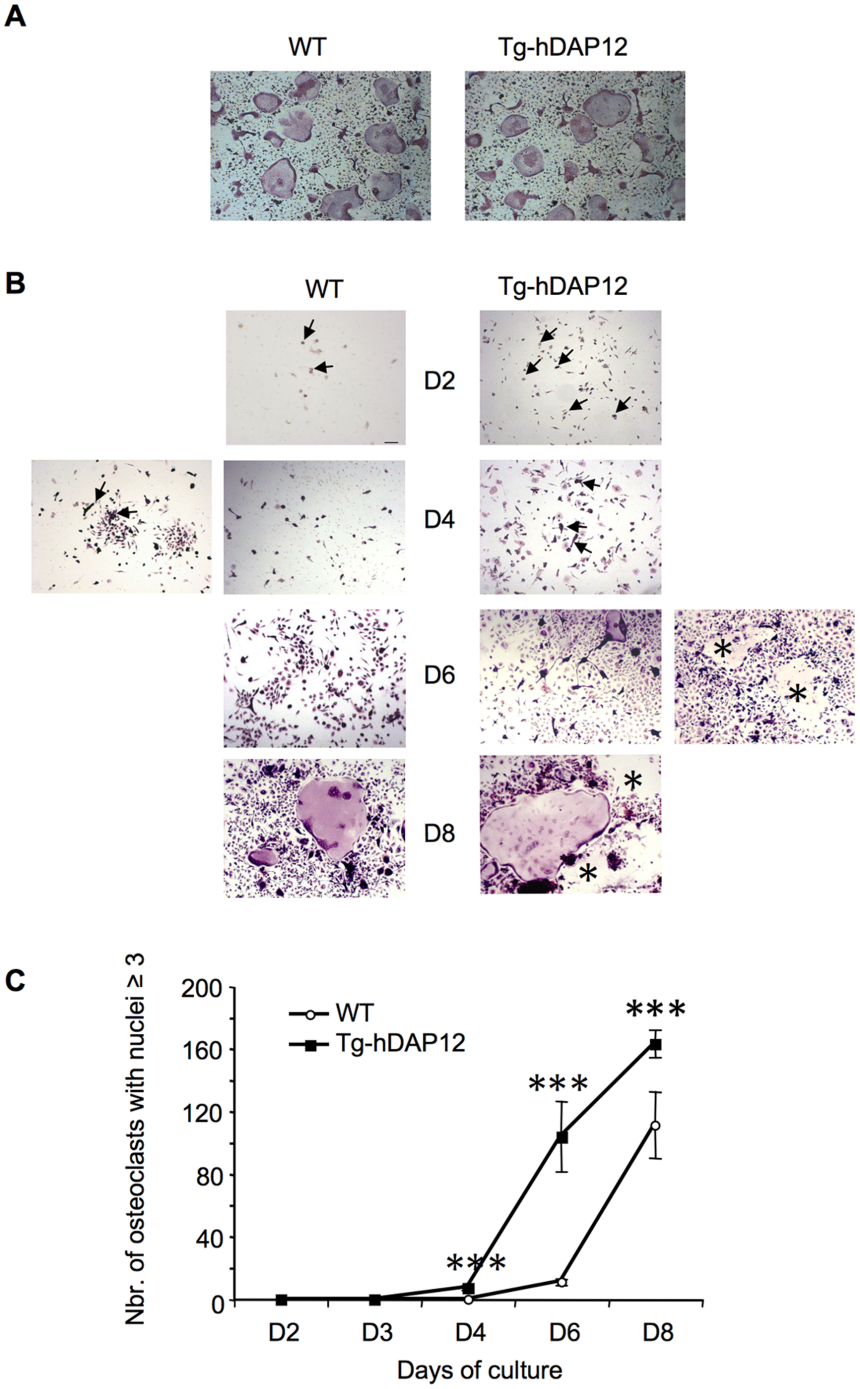
Osteoclastogenesis from bone marrow and spleen cells cultures from 3-month-old Tg-hDAP12 and WT female mice. (A) Osteoclastogenesis from mononuclear bone marrow cells cultured for five days in the presence of M-CSF and RANKL; cells were seeded at 12×10^3^ cells/well in triplicate in 96-well plates; Scale bar 200 µm. (B and C) Osteoclastogenesis from mononuclear spleen cells cultured in the presence of the two cytokines. Cells were seeded at 78×10^3^ cells/well in triplicate in 96-well plates (B) Kinetic studies of osteoclastogenesis over 8 days. Scale bar 100 µm. Cells were fixed in paraformaldehyde and stained for TRAP activity at each indicated time point of the kinetic. Arrows show osteoclast precursors on day 2 (D2) in cultures of WT and Tg-hDAP12 splenocytes. On day 4 (D4) of WT cultures, the images show two different microscopic fields, with osteoclasts formed in foci of proliferating precursors (image on the left). On day 6 (D6) of Tg-hDAP12 cultures, the images show also two different microscopic fields with dead osteoclasts (*in the image on the right). On day 8 (D8) of Tg-hDAP12 cultures the stars (*) also show lysed osteoclasts. One experiment is shown, representative of six independent experiments. (C) Quantifications of TRAP-positive multinucleated cells present in spleen cell cultures at each time point of the kinetic were done on two experiments. Results are mean ± SE.

Bone phenotype indicated an osteopenia in 3-month-old Tg-hDAP12 female mice due to an increased number of osteoclasts, although the level of OPG was increased in the sera of the transgenic mice. We thus investigated *in vitro* osteoclastogenesis with cultures derived from bone marrow and spleen hematopoietic tissues from 3-month-old WT and Tg-hDAP12 female mice in the presence of M-CSF and RANKL. Wild type and Tg-hDAP12 derived bone marrow cultures gave rise to osteoclasts at a similar rate within 5 days (Fig. 3, A). However, osteoclastogenesis from spleen cells was consistently more rapid for Tg-hDAP12 than for WT cultures, as shown by kinetic studies (Fig. 3, B and C). Indeed, after 2 days in presence of M-CSF and RANKL, cell numbers were completely different in WT and Tg-hDAP12 splenic cultures. Many well-spread and evenly scattered isolated mononucleated TRAP-positive cells were visible in cultures of splenocytes from Tg-hDAP12 mice in comparison with few TRAP-positive cells observed in the cultures of WT splenocytes (arrows in Fig. 3, B; D2) indicating accelerated osteoclastogenesis in the culture of Tg-hDAP12 cells. On day 4, some small multinucleated TRAP positive cells (three to five nuclei) were already visible in Tg-hDAP12 cultures. At the same time, in the WT cultures very few of these cells were observed except in the few proliferative foci observed (arrowheads in Fig. 3, B; D4). On day 6, numerous TRAP-positive multinucleated Tg-hDAP12 osteoclasts were observed and some of these were already lysed (Fig. 3, B; stars in D6, right panel). In contrast, WT cultures on day 6 were still at the stage of foci of proliferating adherent cells. When mononucleated precursors were still forming new mature osteoclasts on day 8 in WT cultures, many lysed Tg-hDAP12 osteoclasts were observed (Fig. 3, B; stars in D8, right panel). Monitoring the appearance of TRAP positive osteoclasts with three or more nuclei, and counting them at different time points after seeding, allowed us to establish the kinetics of the differentiation of spleen osteoclast precursors (Fig. 3, C). These results indicated that DAP12 overexpression increased the rate of osteoclastogenesis from spleen, but not from the bone marrow. More rapid osteoclastogenesis observed in the spleen cell cultures of Tg-hDAP12 mice, may be explained either by hyperresponsiveness of the osteoclast precursors to RANKL or by a greater abundance of the precursors, or both.

### Higher Numbers of Splenic Monocytic Progenitors in Tg-hDAP12 Mice

**Figure 4:**
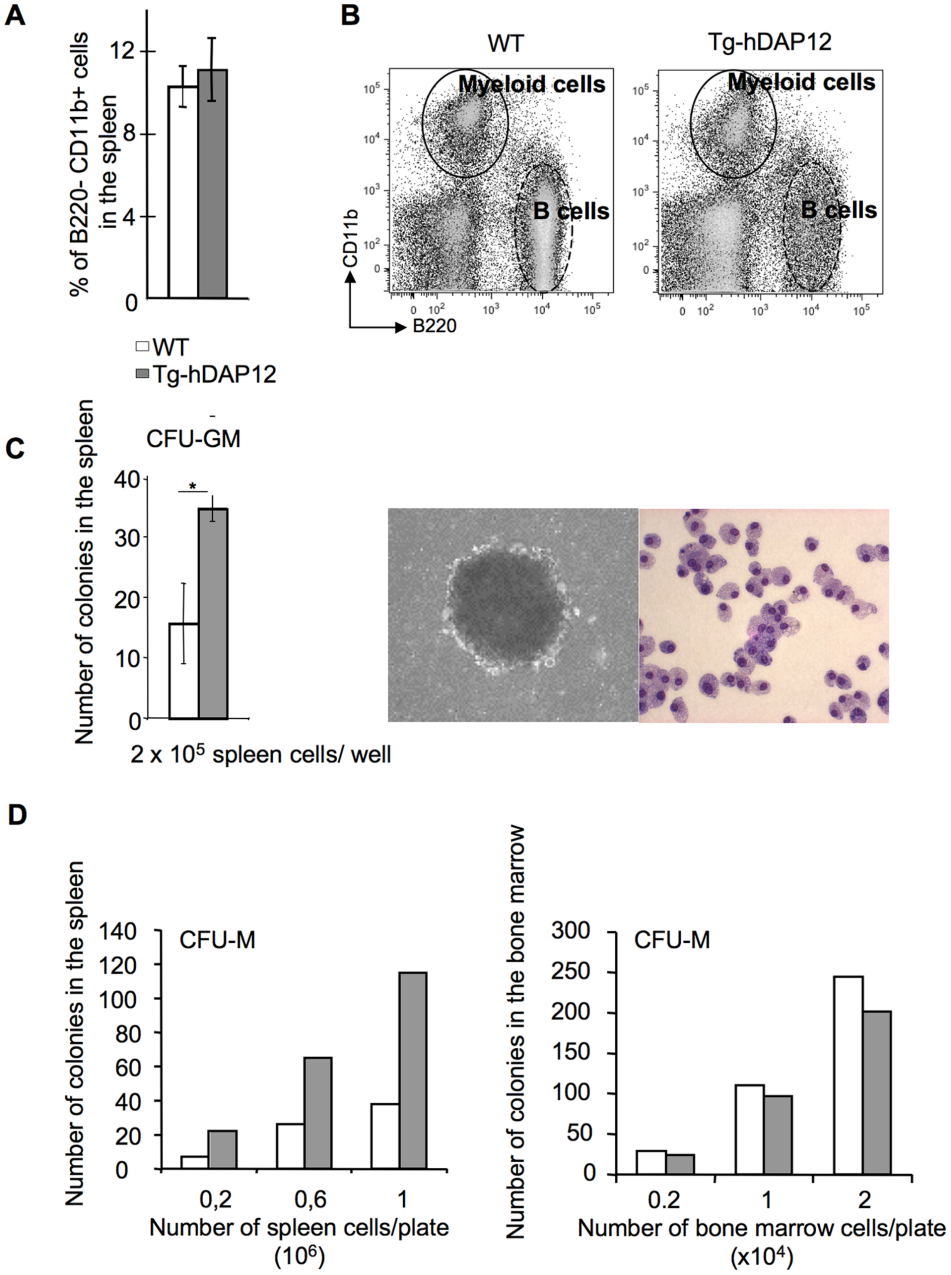
Characterization of monocytic precursors in the spleen of 3-month-old Tg-hDAP12 female **mice.** (A) FACS analyses of myeloid B22O^−^CD11b^+^ cells in the spleen of Tg-hDAP12 mice by comparison with WT mice (four WT and four Tg-hDAP12 animals). The total number of B220^−^CD11b^+^ cells is expressed as a percentage of total nucleated splenocytes. Results are means ± SE. >0.1. (B) One representative FACS dot plot showing the myeloid B220^−^CD11b^+^ population in a black oval and the B220+CD11b–lymphoid population (dashed black oval). (C) Left, Number of CFU-GM colonies obtained after seeding of 2×10^5^ WT or Tg-hDAP12 spleen cells of 3-month-old female mice in Methocult 3534® containing SCF, IL-3 and IL-6 (see Materials and Methods). Results of one representative out of two experiments. Results are means of the number of colonies in the three wells ± SE. *: *p*<0.01. Right, Photographs of one typical colony obtained after 7 days in culture (left picture) and of colony-derived cells after Wright-Giemsa staining. (right picture). The cells have the typical morphology of macrophages. (D) number of CFU-M colonies counted 7 days after seeding WT or Tg-hDAP12 bone marrow or spleen cells in methocult supplemented with 30 ng/ml M-CSF. One experiment performed. Grey bars: Tg-hDAP12 mice; white bars: WT mice.

Since results described so far with Tg-hDAP12 point out to an increased osteoclastogenesis associated to a defect in hematopoiesis especially in the spleen, we performed flow cytometry analyses by using both B220 (CD45R) and CD11b markers. This analyses revealed that there was no statistically significant increase in the classical B220^−^CD11b^+^ myeloid cell population in the spleens of Tg-hDAP12 versus WT, representing 11% and 10% of the splenocytes respectively (Fig. 4, A and dark circle in Fig. 4, B). In contrast, the B cell compartment (B220+ CD11b-) was decreased (dashed black oval in Fig. 4, B).

To test the abundance of osteoclast precursors in the spleen the number of monocytic precursors in the organ of Tg-hDAP12 mice was assessed by performing clonal growth assays. For this purpose, splenic cells depleted of erythrocytes were cultured in semi-solid medium containing promyelocytic cytokines (SCF, IL-3 and IL-6) promoting the growth of CFU-GM progenitors of the granulocyte/macrophage lineages. Interestingly, we observed a two-fold increase in the number of these CFU-GM colonies in Tg-hDAP12 versus WT splenic cells. These colonies were composed of CD11b^+^, Gr-1^+^ B220^−^ cells which were macrophages as shown by their morphology (Fig. 4, C). This study was completed by clonal growth assays in semi-solid medium containing M-CSF. Results showed that there were three times more CFU-M colonies in the spleen of Tg-hDAP12 mice than in WT (Fig. 4, D; left panel) in the same conditions. We noticed an identical number of CFU-M colonies in the bone marrow of WT and Tg-hDAP12 mice (Fig. 4, D; right panel). This clearly indicated that accelerated Tg-hDAP12 osteoclastogenesis observed in spleen cultures was at least partly due to an increase of the starting number of monocytic progenitors (CFU-GM and CFU-M).

### Higher Proliferation of Spleen Osteoclast Precursors in Tg-hDAP12 Mice *in vitro*

**Figure 5:**
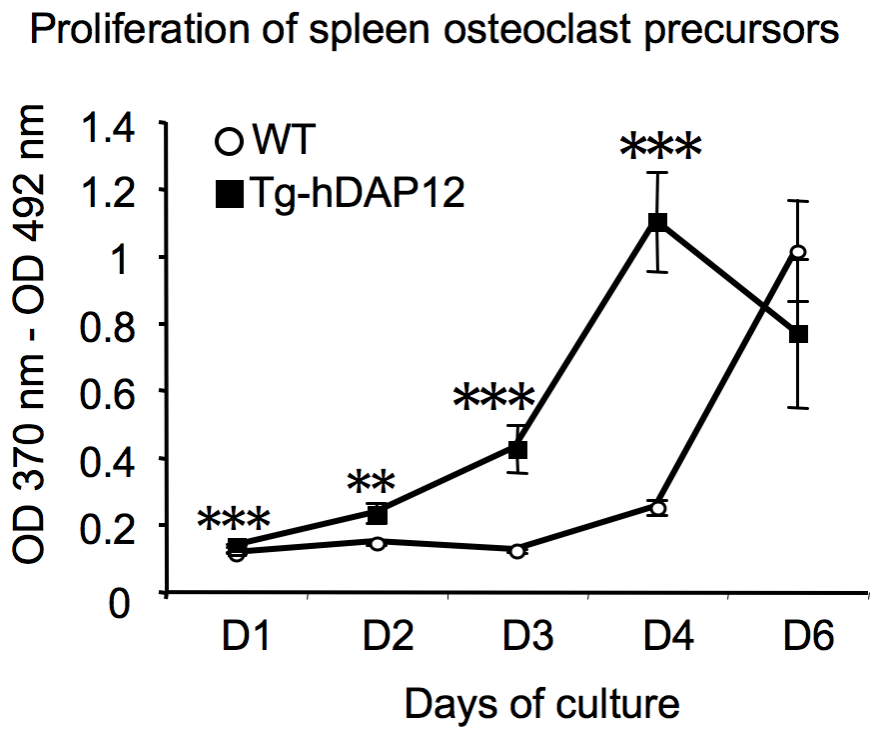
Proliferation of spleen and bone marrow osteoclast precursors. Spleen cells of three 3-month-old WT and Tg-hDAP12 mice were seeded in triplicate in wells of 96-well plates at 78×10^3^ cells/well. At each time point, cells were incubated with BrdU during 3 hours. Incorporated BrdU was measured at 370 nm with a 492 nm reference wavelenght, using a colorimetric immunoassay. White circles: WT cells, black squares: Tg-hDAP12 cells. Results are means ± SE. *p*<0,001 (***);*p*<0,01 (**).

Testing the abundance of the osteoclastic precursors implies also to measure their proliferation rate. Following BrdU incorporation during six days after cell plating in the presence of M-CSF and RANKL showed that the Tg-hDAP12 spleen precursors had a higher proliferation rate than their WT counterparts (Fig. 5). In WT cell cultures the proliferation did not change and was maintained at a low level during the first three days of culture. The WT precursors began to proliferate between day 3 and day 4. At day 4, the proliferation strongly increased to reach a peak at day 6. In contrast Tg-hDAP12 precursors began to proliferate earlier, between day 1 and day 2. The increase of proliferation was observed already between day 2 and day 3, with an important raise between day 3 and day 4, reaching a peak of proliferation at day 4. At this time, differentiation of the first osteoclasts occured, associated with a decreased proliferation, as expected.

### No Hyperresponsiveness of Bone Marrow and Spleen Osteoclast Precursors to Increasing Concentrations of RANKL

**Figure 6:**
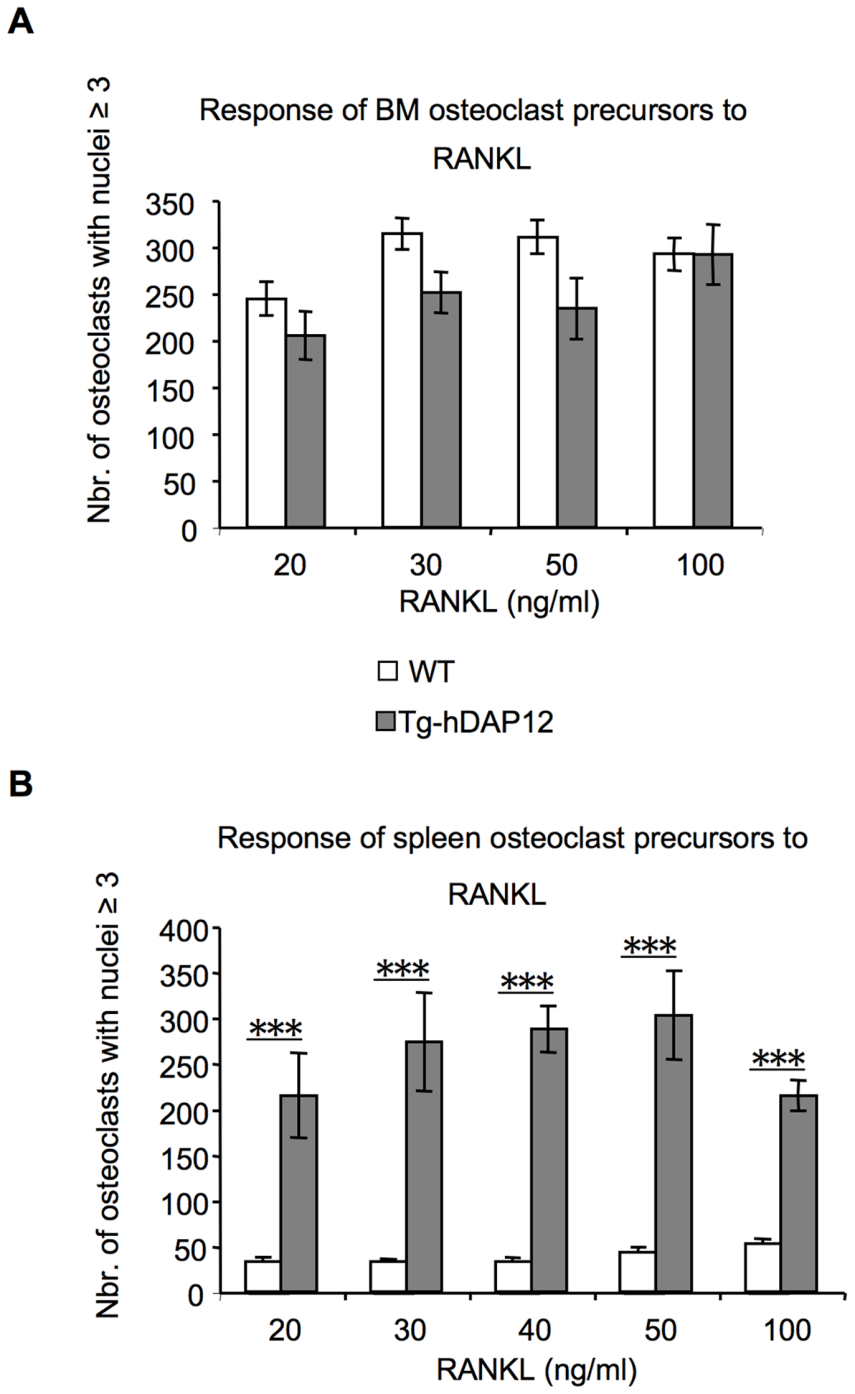
RANKL dose-dependent response of spleen and bone marrow osteoclast precursors. Bone marrow and spleen cells of three 3-month-old WT and Tg-hDAP12 mice were seeded in triplicate in wells of 96-well plates. 12,5×10^3^ cells/well for bone marrow cells and 78×10^3 ^per well for spleen cells. Bone marrow and spleen cells were grown in presence of M-CSF and increasing concentrations of RANKL either 20, 30, 50 and 100 ng/ml for bone marrow cells (A) or 20, 30, 40, 50 and 100 ng/ml for spleen cells (B). After 4 days in culture for bone marrow cells or 6 days in culture for spleen cells, TRAP-positive osteoclasts with ≥3 nuclei were counted. White bars:WT cells; Grey bars:Tg-hDAP12 cells. Results are expressed as mean number of osteoclasts with nuclei ≥3 present on the total surface of each of the three wells ± SE. In (B), ***indicated *p*<0.001.

In order to know whether Tg-hDAP12 spleen and bone marrow precursors were hyperresponsive to RANKL, spleen and bone marrow cells from 3-month-old Tg-hDAP12 and WT female mice were plated with M-CSF and increasing concentrations of RANKL. WT spleen cells were plated at a density three times less than Tg-hDAP12 spleen cells to take into account the difference in the number of monocytic progenitors that we observed in semi-solid cultures assays. Osteoclasts with more than three nuclei were then enumerated in spleen and bone marrow cell cultures respectively six days and four days after cell plating. The response of osteoclast precursors from bone marrow of Tg-hDAP12 to increasing concentrations of RANKL between 20 and 100 ng/ml of the cytokine was not statistically different from that of their WT counterparts. The maximum of osteoclasts (200–300) was already obtained at 20 ng/ml RANKL for both WT and Tg-hDAP12 bone marrow precursors (Fig. 6, A). For spleen precursors of both WT and Tg-hDAP12 mice, the number of osteoclasts did not increase between 20 and 100 ng/ml RANKL (*p*>0.05). In both cases the maximum of osteoclasts was already generated at 20 ng/ml RANKL (Fig. 6, B). The high number of osteoclasts generated in the culture of Tg-hDAP12 spleen cells in comparison with the number of osteoclasts formed in spleen WT cultures results from the presence of a higher number of proliferating monocytic progenitors in the spleen of the transgenic mice (as suggested by our previously described results) and not to an hyperresponsiveness of these cells to RANKL. These data indicated that Tg-hDAP12 spleen and bone marrow osteoclasts precursors were not hyperresponsive to RANKL in comparison with their WT counterparts. In WT spleen cultures, the low number of osteoclasts (30–50) formed at any concentrations of RANKL is probably due to the low number of osteoclasts precursors present in spleen in comparison with their number in bone marrow as it is known in normal WT animals.

### Premature Arrest of B lymphocytes Development at the Pre-ProB/Pre-B Stage

**Figure 7:**
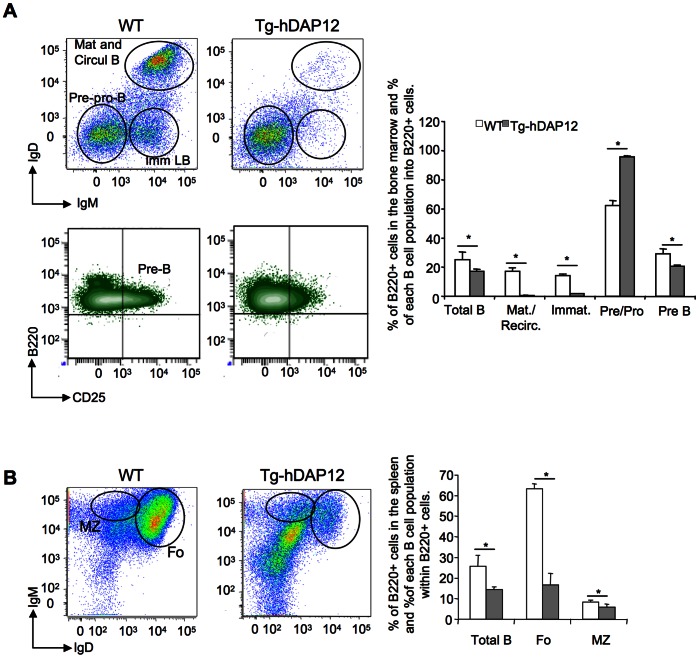
Arrest of B cells development in the bone marrow and effect on spleen B cells populations. Dot plots are representative of independent FACS analyses performed on four WT and four Tg-hDAP12 mice. Histograms represent the mean values obtained for the four WT and the four Tg-hDAP12 mice. (A) B lymphocytes development in the bone marrow. B lymphocytes were first gated as B220^+^ CD19^+^ cells. They were then separated into IgD^+^ IgM^+^ mature and circulating B lymphocytes; IgD^−^ IgM^+^ immature B lymphocytes and IgD^−^ IgM^−^ Pre-and Pro-B cells. In the Pre-and Pro-B cell population, discrimination of Pre-B cells was carried out using labeling of the cells with CD25. The diagram shows the percentage of each B cell population, mature and circulating lymphocytes (Mat and Circul B), immature lymphocytes (ImmLB), Pre/ProB, PreB) within total B220^+^ CD19^+^ cells (Total B). For all populations *p<0.05. (B) B lymphocytes development in the spleen. Cells were first gated as B220^+^ cells. They were then separated in IgD^high^/IgM^low^ follicular lymphocytes (Fo) and IgD^low^/IgM^high^ lymphocytes of the marginal zone (MZ). The diagram shows the percentage of each B cell population (follicular lymphocytes and lymphocytes of the marginal zone) within total B220^+^ cells (Total B). For all populations *p<0.05.

An important decrease in pre-B colonies was previously observed when bone marrow cells of Tg-hDAP12 were cultured in semi- solid culture conditions [26] and was confirmed in our results presented above (Fig. 7, A and B). Indeed, the FACS analyses performed on the cells of these two organs revealed a decrease in the B220^+^ positive cells, (respectively 8% in the bone marrow and 11% in the spleen) comparing Tg-hDAP12 and WT mice (Total B in histograms in Fig. 7, A and B). More interestingly, an important accumulation of Pre-ProB cells was observed in the bone marrow of Tg-hDAP12 mice resulting in 33% more of these cells among the B220^+^ cells of the transgenic animals. This was accompanied by an 8% decrease of Pre-B cells and strong reductions of the percentages of both immature (8-fold) and mature circulating B cells (34-fold) among B220^+^ cells (Fig. 7, A). This impaired B lymphocyte development in the bone marrow resulted in an important decrease in follicular B cells in the spleen of Tg-hDAP12 mice in which they represented 63.3% of B220^+^ cells versus 16.7% in the spleen of WT mice. Marginal zone B cells in the Tg-hDAP12 spleen were reduced to a lesser extent, representing 8.5% of B220^+^ cells versus 6% in the WT spleen (Fig. 7, B). All these data clearly showed that the overexpression of DAP12 resulted in the arrest of B lymphocytes development at the Pre-Pro-B/Pre-B stages in bone marrow and a concomitant increase in myeloid progenitors in spleen.

### Inhibition of Osteoclastogenesis in Co-cultures of Tg-hDAP12 Bone Marrow Osteoblasts with Spleen Osteoclasts Precursors

**Figure 8:**
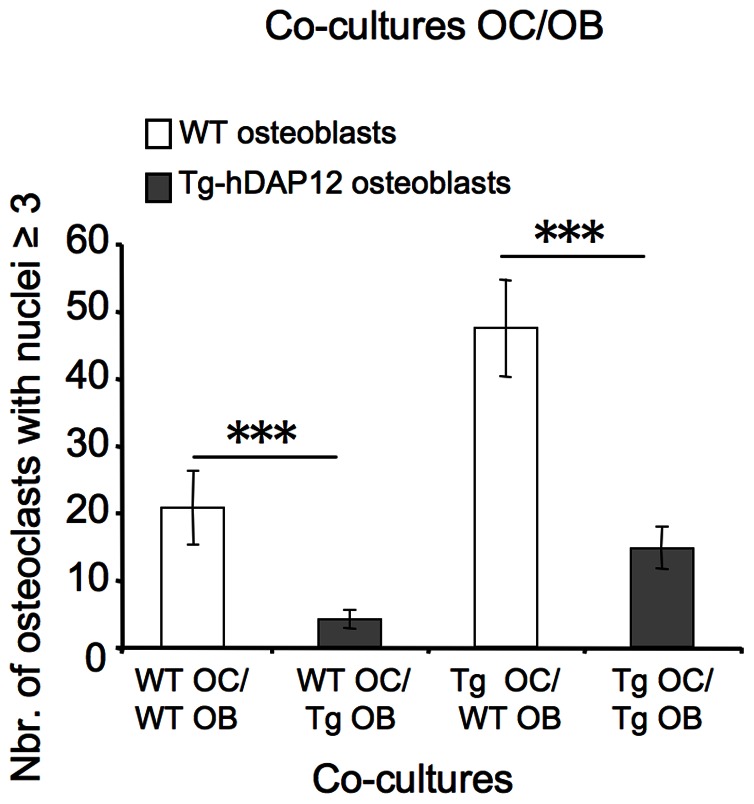
Ability of Tg-hDAP12 Bone Marrow-Derived Osteoblasts to Promote Osteoclastogenesis. Four conditions of co-culture were tested: WT bone-marrow-derived osteoblasts co-cultured with either WT or Tg-hDAP12 spleen cells (respectively WTOC/WTOB, TgOC/WTOB) and Tg-hDAP12 bone marrow-derived osteoblasts co-cultured with either WT or Tg-hDAP12 spleen cells (respectively WTOC/TgOB and TgOC/TgOB). 35×10^3 ^bone marrow-derived osteoblasts of either 3-month-old WT or Tg-hDAP12 female mice were co-cultured with 12×10^5^ cells, isolated from the spleen of 3-month-old WT or Tg-hDAP12 mice. After 6 days of co-culture in presence of ascorbic acid and 1α,25-dihydroxyvitamin D3 at 10^−8^ M, osteoclasts with nuclei ≥3 were counted after fixation and TRAP staining. Results are expressed as mean values of three independent experiments ± SE. ***p<0.001.

Our *in vivo* and *in vitro* previous studies suggested that the differentiation and the biological functions of Tg-hDAP12 osteoblasts were not affected by the overexpression of DAP12. However, we decided to perform co-cultures of WT or Tg-hDAP12 bone marrow osteoblasts with either WT or Tg-hDAP12 spleen osteoclast precursors in order to test the effect of DAP12 overexpression on the ability of Tg-hDAP12 osteoblasts to support osteoclastogenesis in comparison with that of WT osteoblasts. As shown in Figure 8, Tg-hDAP12 bone marrow osteoblasts inhibited osteoclastogenesis independently from the type of spleen osteoclasts precursors with which they were co-cultured (grey bars in figure 8). As expected, a higher number of osteoclasts was present when WT bone marrow osteoblasts were co-cultured with Tg-hDAP12 spleen osteoclast precursors than when co-cultured with WT spleen osteoclast precursors due to a higher number of Tg-hDAP12 spleen osteoclast precursors present in the co-culture (white bars in figure 8).

## Discussion

The non-targeted overexpression of DAP12 has probably many effects on the physiology of the transgenic mice. In this report we have focused our studies on its impact mainly on the bone tissue. However, this study led us to observe hematopoietic defaults in these animals.

We have shown that DAP12 overexpression leads to an osteopenic bone phenotype in contrast to the osteopetrotic bone phenotype described in DAP12 deficient mice [20], [12], [22]. Osteopenia in Tg-hDAP12 mice is characterized by decreases in both number and thickness of trabeculae, as well as increases in the space between trabeculae, and decreases in the cortical thickness, with no change in the bone formation rate. *In vitro,* we observed similar number of mineralized colonies of osteoblasts obtained from Tg-hDAP12 and WT bone marrow cells, cultured in osteogenic conditions, as well as similar patterns of alkaline phosphatase and osteocalcin expressions in these cultures. This has confirmed, that both osteoblastogenesis and bone forming activities of the osteoblasts are normal in Tg-hDAP12 mice. This also suggests that *in vivo* osteopenia is not related to a defect in osteoblastogenesis or in the bone forming function of the osteoblasts. In contrast, this osteopenia is associated with enhanced DPD excretion in the urines of the transgenic animals, suggesting higher bone degradation, not compensated by bone formation activity. Osteoclast-dependent osteopenia is the consequence of an increased number of osteoclasts on the surface of both trabecular and cortical bone. This osteopenia is a slow process, developing progressively over time. The difference in the onset and the severity of osteopenia between Tg-hDAP12 female and male mice can be due to intrinsic gender difference in bone metabolism and is amplified by the hormonal context in the females, since its onset happened earlier during the time course of the female development, at the time of sexual maturity, and results in a stronger osteopenia than in the male mice.

In vitro, osteoclast formation from both WT and Tg-hDAP12 bone marrow precursors is similar in terms of number of generated osteoclasts and rate of differentiation. In contrast, we noticed an accelerated osteoclastogenesis from Tg-hDAP12 spleen precursors reaching a maximum two days earlier than in WT in cultures. Cultures of Tg-hDAP12 spleen cells in semi-solid medium indicated an accumulation of monocytic progenitors (CFU-GM, 2-fold and CFU-M 3-fold) in this organ. The presence of such highly proliferating progenitors explains the high proliferation rate of cultured Tg-hDAP12 spleen cells in comparison with that of their WT counterparts. In Tg-hDAP12 spleen cultures the critical density at which osteoclast precursors begin to fuse is reached earlier than in WT cultures due to the presence of an important number of highly proliferating monocytic progenitors. Accumulation of monocytic progenitors in the spleen of transgenic mice, may result from their egress from the bone marrow into the circulating blood, then reaching the spleen. This enhanced mobilization of the progenitors may be promoted by the increased number of osteoclasts present at the surface of both trabecular and cortical bone [30] [31] or by a modification of the bone marrow microenvironment (hematopoietic niches) or both.

It is difficult to link the osteopenia and the enhanced number of osteoclasts observed *in vivo* only to an enhanced number of splenic monocytic progenitors (CFU-GM and CFU-M) for two reasons: they do not increase in the bone marrow of Tg-hDAP12 mice ([26] and our results) and they represent only 0.01% of splenic cells as calculated from results in figure 4; their increase do not result in a consecutive increase of other myeloid cells than osteoclasts. Thus, it seems that DAP12 overexpression more probably promotes the accumulation of CFU-GM/CFU-M in the spleen. However, we cannot completely rule out the possibility that spleen CFU-GM and/or CFU-M participate in the generation of the osteoclasts in Tg-hDAP12 mice as suggested from the recent work of Kotani et al. [32]This raises the question of the respective relative contributions of bone marrow and spleen to the osteoclast precursor pool, which is not yet known, either in a normal or a pathological context.

This increased Tg-hDAP12 osteoclastogenesis observed *in vivo* is not associated with hyperresponsiveness of both bone marrow and spleen precursors to RANKL *in vitro,* since we have shown that Tg-hDAP12 precursors respond to increasing concentrations of RANKL similarly with their WT counterparts. These data indicated that the effects of DAP12 overexpression on osteoclastogenesis did not result from alterations of responses of osteoclasts precursors to RANKL. Very interestingly, co-cultures of bone marrow-derived osteoblasts and spleen cells clearly showed that bone marrow-derived Tg-hDAP12 osteoblasts inhibit osteoclastogenesis either in presence of WT or Tg-hDAP12 spleen cells. These data are in complete disagreement with the *in vivo* situation. They show that although the differentiation and bone formation activities of Tg-hDAP12 osteoblasts are not affected by DAP12 overexpression, their capacity to support osteoclastogenesis is altered. Since the level of OPG was higher in the sera of WT and transgenic animals, these results suggest that in Tg-hDAP12 osteoblasts the balance between RANKL and OPG syntheses is modified to the advantage of OPG. They also suggest that *in vivo,* at normal serum concentrations of RANKL, some receptors and their associated ligands are able to engage DAP12 downstream signaling. This signaling is strong enough to overcome the osteoblast-mediated inhibition of osteoclastogenesis. In absence of mature B lymphocytes which account for a significant proportion of OPG in bone microenvironment [33], our data suggest that OPG in the bone of Tg-hDAP12 mice is synthesized by other cell sources, at least by osteoblasts. We must also consider that increased levels of G-CSF and TNF-α which have been described in the sera of the transgenic mice [26] may further potentiate osteoclastogenesis**.** The other point of this report is the effect of the overexpression of DAP12 on hematopoiesis suggesting, as mentioned above, possible disturbances of the bone marrow microenvironment in these transgenic animals. From this point of view, inhibition of osteoclastogenesis induced in co-culture by Tg-hDAP12 bone-marrow-derived osteoblasts is an exemple. In favour of this assumption is the block in early B lymphopoiesis in Tg-hDAP12 mice. B lymphocyte development is blocked at a very early stage (Pre-ProB to PreB) in the bone marrow leading to the depletion in follicular B cells and to a lesser extent to that of the B lymphocytes of the marginal zone in the spleen. Furthermore, reports have shown that the expression levels of NFAT transcription factors are down regulated during the comitment of CD34^+^ hematopoietic stem cells and progenitors cells to differentiated myeloid cells [34] [35]; more recently Müller et al. have shown that the expression of an hyperactivable form of NFAT1 (NFATc2) has severe effects on hematopoietic progenitor cell function during hematopoietic and embryonic development [36]. It seems that these effects are related to disturbances of the microenvironment [37] although molecular mechanisms are not known. We can imagine that DAP12 overexpression in the bone marrow microenvironment leads to strong amplification of signals targeting members of NFATs' family. Their activation induces modification of the stroma and of the homing of monocytic progenitors. Ultimately, this results in accumulation of these precursors in the spleen and arrest of B cell development in the bone marrow in which each steps of B lymphopoiesis depends on stage-appropriate niches [38] [39].

We cannot exclude that DAP12 overexpression exerts its effect on hematopoiesis and osteoclastogenesis in a specific independent manner depending on the cell lineage. In the context of osteoclastogenesis, overexpression of DAP12 is similar to the knock out of the SHIP1 phosphatase (Src homology 2(SH2) domain-containing inositol-5- phosphatase): osteoporosis [40] and impaired development of early B cells [41], [42], large increase in CFU-GM cells in the spleen but not in the bone marrow, massive infiltration of macrophages in the lungs in absence of microbial challenge [43]. This infiltration of macrophages has also been observed in Tg-hDAP12 mice with thirty inserted copies of the transgene [26]. It has been postulated that SHIP 1 is a negative regulator of responses of both primitive and mature hematopoietic cells to external stimuli. Furthermore, a recent article has shown that SHIP1 is part of the DAP12 signaling pathway by inhibiting the activation of PI3K mediated by TREM-2 and DAP12 [44]. Further work will be thus needed to study whether SHIP1 and/or Tec kinases can be some of the targets of the DAP12 overexpression and how.
